# The Emerging Role of Silk Fibroin for the Development of Novel Drug Delivery Systems

**DOI:** 10.3390/biomimetics9050295

**Published:** 2024-05-15

**Authors:** Mauro Pollini, Federica Paladini

**Affiliations:** 1 Department of Experimental Medicine, University of Salento, Via Monteroni, 73100 Lecce, Italy; 2 Caresilk S.r.l.s., Via Monteroni c/o Technological District DHITECH, 73100 Lecce, Italy

**Keywords:** drug delivery, silk fibroin, biomaterials, biopolymers

## Abstract

In order to reduce the toxicological impact on healthy cells and to improve the therapeutic response, many drug delivery systems have been fabricated and analysed, involving the use of different natural and synthetic materials at macro-, micro- and nanoscales. Among the natural materials which have demonstrated a huge potential for the development of effective drug delivery systems, silk fibroin has emerged for its excellent biological properties and for the possibility to be processed in a wide range of forms, which can be compliant with multiple active molecules and pharmaceutical ingredients for the treatment of various diseases. This review aims at presenting silk fibroin as an interesting biopolymer for applications in drug delivery systems, exploring the results obtained in recent works in terms of technological progress and effectiveness in vitro and in vivo.

## 1. Introduction

In the modern era of personalized medicine, great attention has been afforded to the potential unwanted effects of a drug that can significantly vary individually, limiting the efficacy of a therapy. Indeed, conventional administration routes, such as tablets, injections and capsules, can result in a major distribution of the drug molecules into different sites from the target site [1], thus resulting in poor bioavailability, fluctuation in plasma drug level and reduced capability to achieve a sustained release [2]. The targeted delivery of molecules to the specific site and at a controlled rate is a crucial aspect in the whole therapeutic process for achieving maximum efficacy and safety without toxicological impact on healthy cells [2,3]. 

Drug delivery systems (DDSs) are designed to provide an efficient transport of the therapeutic agent to the cellular target, through improved aqueous solubility, chemical stability and higher pharmacological activity [4,5]. The selection of drug carriers, additives and processing is also important for the safe and effective transport of the drug molecules [6]. 

In this regard, many options have been explored over the years, and in the past decades huge progress has been made in drug delivery systems based on macro-, nanoscaled and intelligent deliveries [2]. Since the 1950s, when the first controlled release formulation based on the 12 h delivery of dextroamphetamine (Dexedrine) was introduced by Smith, Kline and French, many other options have been explored [4,7] and, for more than 60 years, numerous DDSs have been developed for helping patients and maximizing drug efficacy [8]. During the first generation of drug delivery systems, developed between 1950 and 1980, oral and transdermal release and the related release mechanisms were investigated [7,8]. Further developments of the drug delivery technologies introduced the use of the so-called “smart polymers” and hydrogels, particularly for the systems triggered by environmental stimuli such as pH, temperature, glucose level, etc. [7], which can determine changes in some properties such as hydrophilicity, swelling, permeability, etc. [9,10]. More recently, the use of bioresponsive systems at a nanometric scale has gained great attention for applications in tissue engineering, imaging and biosensors, due to the interesting capability of the nanocarriers in targeting specific cells and encapsulating a wide range of therapeutic molecules [11,12]. 

Over the years, intense research has been conducted regarding the definition of effective drug delivery systems, through multidisciplinary approaches focused on the development of novel technologies and biomaterials. In this scenario, silk fibroin, a biomaterial extensively studied for tissue engineering, has garnered huge interest for drug delivery applications due to its excellent properties, including versatility in processing and biocompatibility. This review aims to explore some recent and interesting progress achieved in this field involving the use of silk fibroin. 

## 2. Biomaterials for DDSs

Biomaterials play a key role in tissue engineering by mimicking the tissue environment through engineered architectures for cell growth and by providing structural support for the delivery of cells and therapeutic factors [13].

Biomaterials can interact with biological systems for both therapeutic and diagnostic medical purposes, and are also an interesting choice in drug delivery systems for modulating the pharmacokinetics of the drugs. According to the type of the biomaterial and the different scales from nano- to macro-sized, many DDSs with different physico-chemical properties and drug release profiles can be designed [2,14].

The choice of the proper biomaterial should be based on the formulation and on the route for drug delivery; moreover, biocompatibility, biodegradation, good mechanical properties and bioavailability are important criteria for selecting the best option for achieving the desired response [2]. In the last two decades, different polymeric drug delivery systems have been developed, based on natural or synthetic materials and on biodegradable and bio-adsorbable polymers [15]. Both natural and synthetic polymers have been addressed as potential candidates for DDSs [3]. Although synthetic polymers have attracted a significant number of research works, some issues are related to their limited control of structure and in some cases to low biocompatibility [16]. Indeed, although the main advantage of synthetic polymers can be associated with their availability in bulk and the possibility of tailoring their properties, they do however lack cell recognition signals and sometimes their degradation products may be toxic [17]. In contrast, numerous remarkable advantages are associated with biopolymers in the design and fabrication of DDSs, including biodegradability, biocompatibility, availability, versatility and the capability to specifically interact with certain molecules [18,19]. Also, other important advantages of biopolymers are that they have been proven to be non-toxic, non-carcinogenic, non-thrombogenic, easy to extract and, very importantly, they are environmentally friendly, with the least harmful impact on the environment in terms of pollution [18,20]. Hence, the attention towards biopolymers has recently increased and the biopolymer industry has significantly grown, as demonstrated by the increased sales of biopolymers by 20–30% per year [20].

An example of a natural polymer widely studied for designing advanced drug delivery systems, both as a carrier and as an excipient, is nanocellulose, which has demonstrated great potential due to its high surface area-to-volume ratio and high polymerization, which determine the high loading and binding of active pharmaceutical ingredients, along with the control of the drug release [21]. Biopolymers derived from polysaccharides (such as chitosan and hyaluronic acid) and proteins (such as silk fibroin and collagen) are becoming more popular than synthetic polymers [3,19,22,23] and have been investigated for higher therapeutic outcomes and for potential scale-up to clinical level [3,19].

**Figure 1:**
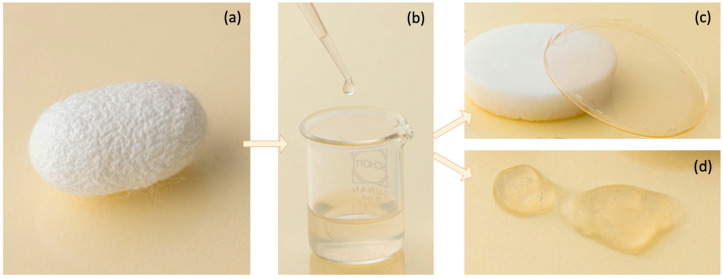
Examples of silk fibroin-based products for drug delivery obtained from silkworm cocoons. Silkworm cocoon (**a**), silk fibroin solution (**b**), three- and bi-dimensional devices ((**c**), left and right, respectively) and fibroin hydrogel (**d**).

In particular, among these interesting biopolymers for DDSs, this review aims to focus attention on silk fibroin, which possesses ideal properties for drug delivery polymers and offers a bottom-up approach that helps to introduce novel features [13,24]. Indeed, silk has demonstrated multiple advantages, such as biocompatibility, biodegradability, non-toxic degradation products, sterilization, mechanical stability and self-assembly properties [17,18,25]. Moreover, by tuning the chemical composition and the physical structure of silk molecules, various formats of silk-based materials, such as hydrogel, films and 3D porous aerogels, and nanofibers/particles and textiles can be derived from the fibroin solution (organic or aqueous) through different processing techniques [18,25,26]. Some examples of silk-based devices that can be used for drug delivery applications are reported in Figure 1 where some products developed by the authors are shown. In particular, through appropriate procedures aimed at preserving the high molecular weight of the protein, from the silkworm cocoons (Figure 1a) the silk fibroin solution can be obtained (Figure 1b). This is the starting point for processing, for example, the protein into three- and bi-dimensional structures (Figure 1c, left and right, respectively) or hydrogel (Figure 1d).

Among these various forms, due to their versatility, hydrogels stand out as promising biological platforms for cell/drug carriers, extracellular matrix-like (ECM-like) scaffolds and tissue fillers. As injectable solutions, in situ gels, structured matrices, viscous gels and thin sheets, they provide important structural and functional support for cell functions and for sustained release of the desired factors [13,26].

## 3. Silk Fibroin for Drug Delivery

Beyond its use in the textile industry, the application of silk as biopolymer has been extended to various high-tech areas due to its outstanding properties that, compared to other synthetic and natural polymers, have allowed for many innovative applications [6]. Silk fibroin, the major component of silk (about 75%) is obtained via a degumming process through which sericin, the outer glue-like protein, is removed [27]. Fibroin has attracted intense research in recent decades, particularly in wound healing, due to the promising results obtained in vitro and in vivo by employing fibroin in different forms [28,29,30,31,32,33]. In addition to skin regeneration, until now, a variety of fibroin-based hydrogels, 3D porous scaffolds and mats have been fabricated for regenerating different tissues, including nerves, tendons, bone and cartilage, ligaments and blood vessels [34,35,36,37,38,39,40].

Moreover, the advances in processing technologies have allowed for more options for combining and functionalizing silk fibroin with other biomaterials for some specific purposes [41,42,43]. Over the last 20 years, silk fibroin has inspired almost 10,000 published works in tissue engineering and has been adopted to develop a wide range of materials with tailored properties and architectures for specific applications [44]. Indeed, silk fibroin (SF) produced by Bombyx mori has been recognized as one of the most interesting biomaterials for its excellent features such as biocompatibility, tunable biodegradability, mechanical strength and bioactivity, that have inspired huge scientific progress in the medical field, such as tissue engineering, regeneration of soft and hard tissues, cancer therapies and controlled drug delivery [45,46]. The possibility of processing silk fibroin into multiscale dimensions, such as nano- and micro-particles, hydrogels and thin films, has made this protein an ideal candidate for drug and gene delivery. Silk devices for drug delivery have been generally developed through the incorporation or dissolution of the drug into a silk fibroin solution, then processed into different forms, where the molecular weight of the drug and its interaction with silk is crucial to determine the release kinetics [47].

The mechanism of binding drugs to silk fibroin and the microstructure of silk play an important role in drug release. It is reported that strong electrostatic binding between silk and bound molecules can avoid significant burst release, and an increase in β-sheet content in protein structure is responsible for slowing down the release rate [48].

A conformational transition from random coil to β-sheet structure, which results in a physical crosslinking, can be induced in fibroin by physical factors such as the pH, temperature, solvents, cations and vortexing. The physical crosslinking is very promising for biomedical applications due to the low costs, safety and the absence of toxic chemical agents, whilst chemical crosslinking involves photo-polymerization or chemical and enzymatic crosslinking agents [26,27,49]. The capability of the silk fibroin chains to form physical networks through gelation mechanisms has been widely explored in the last decade for the preparation of self-assembled silk fibroin hydrogels [50]. Although the physical gelation mechanisms of fibroin still require deeper studies, silk fibroin hydrogels have emerged as a good option for cell culture, tissue engineering and controlled drug delivery [26,49,50,51]. They have also been considered as pH-responsive drug delivery materials, with a release profile that can be controlled by the molecular weight of the protein [13]. Moreover, the design and control of the multi-scale structure of silk fibroin have allowed for the development of hydrogels with high strength, injectability, self-healing, adhesive properties, stimuli responsive capability, etc. [52].

Different successful clinical studies on silk-based delivery systems have encouraged an increasing number of applications for the protein-based delivery of drugs, genes and other molecules [53]. Recently, fibroin nanoparticles have been proposed for the encapsulation of different therapeutic compounds, such as proteins, enzymes and vaccines. Along with their versatility and the possibility to be chemically modified, fibroin nanoparticles can be administrated via parenteral, oral, transdermal, ocular, orthopedic and respiratory routes [24]. In form of nanofibers, many advantages for the development of drug delivery platforms are associated with high surface area, mechanical properties, surface modification and the incorporation of functional moieties for enhanced bioactivity. In this regard, an electrospinning technique applied to silk fibroin has been adopted for producing nanofibers for the delivery of various types of biomolecules [24]. Another technique for the delivery of drugs to the site rather than oral and injectable administration is based on the use of transdermal microneedles. Silk fibroin microneedles have been deeply explored for increasing the drug doses and types, and for developing systems with stimuli-responsive release functions [54].

## 4. Applications of Silk Fibroin in DDSs

The skin epidermis, comprising five anatomical layers with the stratum corneum as outermost layer, is considered the best organ for drug delivery [55]. Easy administration, bypassing the first-pass metabolisms, the large skin surface, the low costs and patient compliance are some examples of the advantages related to transdermal drug delivery [56]. On the other hand, the skin barrier represents an obstacle for drugs, namely the stratum corneum [55,57]. After permeation through the stratum corneum into the interfollicular region, the drugs can diffuse through the diffusion process towards the target organ [55]. To overcome this excellent skin barrier, microneedle-based devices have emerged in transdermal drug delivery, also providing efficient and patient-friendly methods for delivering different molecules and compounds [58]. The microneedles are micrometric-sized needles (50–900 μm in length) in arrays that can successfully penetrate the stratum corneum creating reversible microchannels in a minimally invasive manner, and deliver drugs without involving blood vessels and nerves in the dermis [57,59]. Due to its bioavailability, non-invasive treatment process, controlled drug release and robust plasma drug screening, transdermal drug delivery can be used in many fields, including the treatment of cancer, obesity, arthritis, and soft tissue engineering [60]. The microneedles can be fabricated in different forms, such as solid, dissolving and hydrogel-based microneedles [61], nanoparticle loaded/coated polymeric microneedles, aiming at reducing the side effects and administration frequency to improve patient compliance [62], offering the opportunity to target the delivery of drugs and vaccines to specific tissues [63]. The geometry for the manufacturing, the selection of the proper material for microneedles, the amount of drug loading and the potential skin reaction are some challenging aspects that require great attention [64]. Polymeric microneedles are interesting transdermal drug delivery systems because of the controlled degradation and swelling profile and the diffusion profile of the encapsulated drug [65,66]. A range of macromolecular compounds have been adopted for the fabrication of microneedles, including water soluble sugars, biodegradable polymers, carboxymethlcellulose, hyaluronic acid and silk fibroin [58].

In particular, natural polymers, such as silk fibroin, have been used in reverse polydimethylsiloxane (PDMS) fabrication molds and have demonstrated the ability to swell to aid drug release, biocompatibility and strength [58]. Yin et al. presented a swelling-modified silk fibroin microneedle for transdermal delivery. A composite comprising 2-ethoxyethanol and silk fibroin was used to fabricate the silk fibroin-based microneedle systems by pouring the solution in a polydimethylsiloxane (PDMS) mold and obtaining a solid microchannel array. The system was proposed to pierce porcine skin in a dried state with a depth of ∼200 μm, and to swell to a hydrogel state through the absorption of interstitial fluid. Better swelling properties, which determine larger pores, are associated with higher transdermal drug release kinetics, and a relation between pore size and molecular weight of the encapsulated therapeutic agent can also be determined [67].

Yavuz et al. reported the fabrication of silk-based microneedles patches for sustained release of a synthetic progestin contraceptive, namely levonorgestrel, as a new option for women against unwanted pregnancy. The formulation of silk and the drug loading were modified to tune the release profile over the time and, for further control, different molecular weights of the silk were tested. The formulation of all the microneedles developed by the authors demonstrated a drug delivery capability above the required daily contraceptive dosage of 30 μg/day [62].

Qi et al. developed a melatonin-loaded silk fibroin microneedle patch for the treatment of insomnia and tested the system in vivo on a Sprague Dawley (SD) rat model with insomnia. The drug-loaded microneedles, produced with a uniform shape and with the necessary strength for penetrating the skin without breaking, were mainly made of crystal Silk I with a low dissolution rate and suitable swelling. The results indicated an accelerated time for the entrance of melatonin into the blood, which improved the bioavailability of the drug, also reducing the risk of excessively high concentrations [68].

In the treatment of diabetes, which globally affects about 450 million adults, the management of hyperglycaemia is very important to avoid tissue damage that can develop into fibrosis. The subcutaneous administration of insulin is conventionally adopted which, although effective, has some limitations, such as the pain. The transdermal route through microneedles is a valuable alternative, due to the minimal invasiveness and painless treatment, that can also be used to combine both glucose monitoring and drug delivery in more sophisticated devices [69]. Sakunpongpitiporn et al. demonstrated that silk fibroin hydrogels produced through solution casting can deliver insulin at the required release, amount, and duration, also suggesting that these aspects can be controlled by the matrix fibroin concentration and the electric field in transdermal iontophoresis insulin delivery [70].

Zhu et al. developed a composite insulin-loaded microneedle patch using silk fibroin as raw material. The tip of the fibroin needles provided the mechanical properties and also demonstrated good solubility and biodegradability, thus promoting the release of insulin without rejection in the rats [71]. In their study, Cao et al. demonstrated the excellent therapeutic effect of a silk fibroin-based drug delivery system in the maintenance of a proper insulin activity. The system, based on swellable microneedles for the controlled release of insulin, exhibited advantages in improving application time with larger dosages [72]. In addition to its role in the metabolism of glucose, insulin is involved in many cellular functions, such as cellular growth and differentiation, and the wound healing process, making it an interesting drug for the management of different diseases [73]. Cubayachi et al. have explored the application of insulin in the treatment of corneal wounds by developing silk fibroin films containing insulin for sustained drug release and including glycerin as plasticizer. Transparent and homogeneous insulin-loaded films, prepared by casting, demonstrated effectiveness in the stabilization and release of insulin. The films were tested in vivo on Hannover Wistar rats and a reduction in blood glucose promoted by the samples was observed [73]. The delivery of drugs for corneal diseases is attractive but challenging at the same time, due to the unique anatomy and the barriers of the eye. In addition, many ocular diseases are linked to age-related issues, such as macular degeneration and glaucoma [74], which is the second leading cause in the world for irreversible impaired vision and mainly associated with high intraocular pressure [75]. Silk fibroin hydrogels were proposed by Lovett et al. for the sustained ocular drug delivery of the anti-vascular endothelial growth factor (anti-VEGF) bevacizumab in the treatment of age-related macular degeneration. The sustained release was confirmed for 3 months in a rabbit model, with timespans of hydrogel release at least 3-fold greater than aqueous controls [76].

**Figure 2:**
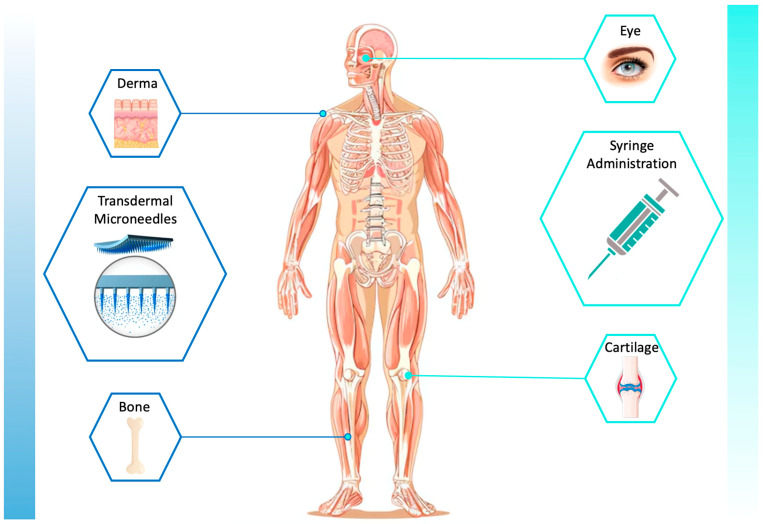
Examples of DDS application fields.

For the treatment of ocular diseases such as glaucoma, age-related macular degeneration, retinal vascular occlusion, etc., the microneedles have been suggested for delivering free or encapsulated drugs with reduced invasiveness by employing polymeric nanocarriers and in situ gels [77]. For example, PVA and PVP were used by Roy et al. as a matrix for producing microneedle ocular patches for delivering pilocarpine, a drug usually employed in the treatment of glaucoma. The patches, produced by micromolding, had the shape of commercial contact lenses [78]. Regenerated silk fibroin films blended with chitosan were developed by Jeencham et al. for disposable therapeutic contact lens-based ophthalmic delivery. The films were loaded with diclofenac sodium, a hydrophilic anti-inflammatory agent, through the soaking method, and drug loading capacity and drug release profile were evaluated in relation to the film fibroin content [79]. In addition to derma and the eye as routes for delivering drugs and therapeutic molecules, important results have been achieved in the application of fibroin-based delivery systems for cartilage and bone [80]. Figure 2 visually summarizes the examples of drug delivery applications presented and reviewed in this article, namely DDSs for derma, eye, cartilage and bone applications.

In cartilage, the use of SF hydrogels has been proposed for embedding drugs to treat chondritis and to accommodate chondrocyte regeneration [81]. Articular cartilage injuries are characterized by a limited self-repair capability due to the shortage of blood vessels, lymphatics and nerves. In their study, Giang Phan et al. designed an injectable hydrogel system, mimicking the extracellular environment and enabling the delivery of an anti-inflammatory drug for cartilage restoration. For this purpose, the authors used silk fibroin and hyaluronic acid as bioinspired materials mimicking the function of living tissues, showing controlled biodegradation after implantation in a mouse model and a loss of only 28% of the hydrogel mass in the first week [80]. In an attempt to mimic the physiology of bone, various morphologies of silk fibroin have been investigated for the delivery of bone specific growth factors (such as BMP-2, platelet-derived growth factors), with a crucial role in promoting osteogenesis for the application in bone regeneration [82]. In the prevention and management of bone-related disorders, despite the progress of the conventional drug delivery systems, the limited drug absorption and lack of specificity in therapeutic outcomes have encouraged the use of microneedle-based therapy as a safe and well-tolerable therapeutic approach [83]. For example, a composite microneedles system consisting of silk fibroin needle tips and a hyaluronic acid base has been developed by Li et al. for the transdermal delivery of salmon calcitonin for the treatment of osteoporosis. After application, the hyaluronic acid base dissolved, allowing for the implantation of SF drug depots into the skin for the controllable sustained release of the drug. Compared with traditional needle injection, this delivery system showed better trabecular bone repair in ovariectomized-induced osteoporosis in mice [84].

## 5. Conclusions and Future Perspectives

Drug delivery systems overcome some issues associated with drugs, among which are issues related to stability, the specific targeting of cells and tissues, stabilization of drugs and control of release rates, solubility, bioavailability, and therapeutic outcomes [5]. Many options, based on both natural and synthetic materials, have been explored for different applications and, among them, drug delivery systems based on silk fibroin protein have attracted great attention because of the many advantages associated with biocompatibility, biodegradability, ease of sterilization, and processability [85].

In this review, interesting progress regarding the use of fibroin in DDSs has been reported and many different delivery systems have been developed by using fibroin as film, hydrogel, or microneedles. Despite the numerous advantages and the promising results obtained in this multidisciplinary topic, there are some aspects of this research that still need to be more deeply investigated, particularly in relation to the limited number of clinical trials [24,86]. For example, although the microneedles have displayed the potential to deliver various molecules, more studies still need to be completed at the preclinical stage, thus bridging the gap between the laboratory and clinical application [62]. The literature has provided insights into the technologies and biopharmaceutical effects, but the translation from preclinic to clinical application is challenging [19] and, for this purpose, an efficient collaboration between academic and industrial players, organizations and hospitals is necessary [62]. An international, non-profit, global health organization named PATH has established the “Microarray Patches-Centre of Excellence” for accelerating both the manufacturing of microneedles and regulatory processes [62].

Silk as a biopolymer has been extensively studied for decades for tissue engineering, but its use in drug delivery applications has emerged more recently. The increasing research on the potential of silk fibroin as active biological polymer will inspire a range of materials that will enter clinical testing and clinical use [87].
